# Impact of the COVID-19 pandemic and its measures on one-year and three-year cancer survival using Swiss cantonal cancer registry data

**DOI:** 10.1007/s10552-026-02175-8

**Published:** 2026-05-26

**Authors:** Flurina Suter, Miriam Wanner, Andreas Wicki, Dimitri Korol, Sabine Rohrmann

**Affiliations:** 1 Division of Chronic Disease Epidemiology, Epidemiology, Biostatistics and Prevention Institute (EBPI), University of Zurich, Zurich, Switzerland; 2 Division of Medical Oncology and Hematology, Cancer Registry Zurich, Zug, Schaffhausen and Schwyz, University Hospital Zurich, Zurich, Switzerland; 3 Department of Medical Oncology and Hematology, Faculty of Medicine, University and University Hospital Zurich, Zurich, Switzerland

**Keywords:** Coronavirus disease 2019, Cancer survival, Counterfactual analysis, Switzerland, Cancer registry

## Abstract

**Purpose:**

The COVID-19 pandemic and associated measures negatively impacted health outcomes of patients with severe diseases. This study investigated the short- and intermediate-term impact of the pandemic on cancer survival.

**Methods:**

This study was based on cancer registry data of the Swiss cantons of Zurich and Zug from 2018 to 2021. Observed and relative one- and three-year survival were calculated based on lifetables for the population covered by the registry. Multivariable Royston Parmar survival regression models were fitted to compare survival before (2018/2019) and during the pandemic (2020, 2021). For patients diagnosed during the pandemic, a counterfactual analysis was conducted. Analyses were performed for overall cancer and the five most common cancer types.

**Results:**

Overall, 32 826 cancer cases were included. One- and three-year survival for all-cancer continuously increased from 2018 to 2021. Compared to pre-pandemic, the hazard of death was lower for all-cancer during the pandemic (2020: Hazard Ratio 0.93 [95% Confidence Interval 0.89, 0.96]; 2021: 0.91 [0.87, 0.94]) and for skin melanoma in 2021 (0.79 [0.62, 0.98]), but higher for colorectal cancer during the pandemic (2020: 1.17 [1.03, 1.32]; 2021: 1.24 [1.09, 1.40]). Observed one-year survival for all-cancer, female breast, and prostate cancer was higher in 2021 than predicted. In 2020, observed one-year survival of colorectal cancer and three-year survival of female breast cancer were lower than predicted.

**Conclusion:**

The COVID-19 pandemic appears to have had only a minimal impact on the overall increasing cancer survival trend in Switzerland, reflecting the robustness and resilience of the healthcare system.

**Supplementary Information:**

The online version contains supplementary material available at https://doi.org/10.1007/s10552-026-02175-8.

## Introduction

End of 2019, the severe acute respiratory syndrome coronavirus 2 was detected and rapidly spread around the globe [1], causing the Coronavirus Disease 2019 (COVID-19) pandemic [2]. Worldwide several pandemic measures were implemented to lower the number of new COVID-19 infections [3]. On March 16, 2020, the federal council declared a state of emergency in Switzerland and mandated a national lockdown in spring 2020 [4]. During the lockdown, the public life of Swiss residents was constrained, and healthcare institutions were restricted to conduct only urgent procedures [5].

Even though the pandemic measures led to a reduction in COVID-19 incidence in Switzerland [6] and globally [7], they have negatively impacted the health outcomes and care of patients with other severe diseases including cancer [8]. Impacts of the pandemic were observed on the whole cancer care trajectory. Globally [9]. and in Switzerland[10] fewer cancer screening tests were performed. In addition, fewer cancer diagnoses [11–13], an upstaging in cancer stage [13–15], shifts in administered cancer treatment and time to treatment initiation [16, 17], and reduced cancer survival [13, 18–20] have been observed.

Two smaller Swiss studies reported an upshifting in cancer stage distribution during the pandemic for sinonasal malignancies [21] and melanoma [22]. Using population-based Swiss cancer registry data, less cancer diagnoses during the lockdown phase [23, 24] and shifts in first cancer treatment during the pandemic were observed [25], but there was no major impact on cancer stage distribution [24, 25] and one-year survival [24].

A gap in knowledge exists regarding the impact of the pandemic on short- and intermediate-term survival of cancer patients in Switzerland, while adjusting for known confounders. Therefore, our study investigated the impact of the pandemic on cancer survival using population-based cantonal cancer registry data of the Swiss cantons of Zurich (ZH) and Zug (ZG) from 2018 to 2021 with follow-up data until 2024.

## Methods

The outline of the REporting of studies Conducted using Observational Routinely-collected Data (RECORD) guidelines was followed [26].

### Cancer registry and study population

This study was based on cancer cases registered at the cantonal cancer registry of the cantons of ZH, ZG, Schaffhausen (SH), and Schwyz (SZ) (CRZZSS) in 2018/2019 (pre-pandemic years), 2020 and 2021 (during pandemic years). The cantons of ZH and ZG started cancer registration in 1980 and 2011, while the cantons of SH and SZ started in 2020. The latter two cantons did not provide data for the pre-pandemic years and were therefore excluded. The study investigators had full access to the data.

In 2020, the Cancer Registration Act came into force in Switzerland, requiring all private and public health institutions to report each cancer case to the responsible cantonal cancer registry [27]. The law imposed a common, complete, and high-quality cancer registration at a national level [27]. Nevertheless, data quality of the CRZZSS has shown to be acceptable and increasing even before the implementation of the law in 2020 [28].

The CRZZSS acquires sociodemographic information on each cancer patient including their age, sex, living region, vital status, and last follow-up date. Details on the cancer are recorded as well, including information on cancer type, grade, TNM classification, stage, and date of diagnosis based on the guidelines provided by the National Agency for Cancer Registration and the Childhood Cancer Registry [29]. Details on the whole cancer registration process are provided online [30].

To classify cancer cases, the International Classification of Diseases (10th revision) (ICD-10) [31] code is used. The TNM classification is based on the TNM classification of malignant tumors, 8th edition, by the Union of International Cancer Control [32], while cancer stage is based on the American Joint Committee on Cancer tumor, node, and metastasis stage classification manual [33]. Cancer cases are categorized as stage I to IV or unknown based on pathological stage information or, if the pathological stage information is unavailable, on clinical stage information.

All registered primary malignant cancer cases (except non-melanoma skin cancer ICD-10 C44) and benign brain tumors (ICD-10 D32—33, D43) were included. The registration of non-melanoma skin cancers was only fully conducted after the implementation of the law in 2020, leading to non-representative data on non-melanoma skin cancer in the pre-pandemic period. For each patient, only the first incident primary cancer diagnosis observed during the study period, 2018—2021 was selected. All cancer diagnoses of patients with multiple first primary cancer cases diagnosed on the same date were excluded. Cases diagnosed based on a Death Certificate Only (DCO) [34] and cases with an inaccurate incidence date were excluded, because their exact survival time was unknown. The remaining cancer cases were defined as all-cancer group. In addition, the five most frequent cancer types in Switzerland were investigated individually: colorectal cancer (ICD-10 C18-C20), lung cancer (C34), prostate cancer (C61), breast cancer (only females; C50), and skin melanoma (C43).

A flowchart of the study population and exclusion criteria is provided in the supplementary materials (Figure S1).

### Statistical analyses

Baseline characteristics of the study population were analyzed descriptively. Categorical variables were shown as absolute numbers and percentages, while continuous variables were shown as median and interquartile range (IQR).

Survival time was defined as the time between cancer diagnosis and death, last follow-up date, or loss of follow-up date, whichever occurred first. Observed one-year and three-year survival were determined by using the Kaplan–Meier method [35]. Observed survival indicates the proportion of cancer patients alive one year and three years after diagnosis. Relative one-year and three-year survival were determined by using sex-specific, annual period lifetables for the population covered by the CRZZSS, assuming a constant annual probability of death at the age of 99 years and beyond, and by applying the Ederer II method [36]. Relative survival compares observed survival among cancer patients to expected survival in the general population, accounting thereby for mortality unrelated to the cancer diagnosis. Observed and relative survival were age-adjusted using the weights for five broad age classes (0—44, 45—54, 55—64, 65—74, 75 + years) from the International Cancer Survival Standard to enable comparisons across time periods and with studies using data from other countries [37]. For the analysis of three-year prostate cancer survival in 2019, the sample size in the youngest age group was too small to calculate an age group-specific survival estimate. Therefore, the two youngest age groups were combined for this specific analysis. Relative survival larger than 100% can occur when observed survival among cancer patients was larger than expected survival in the general population [38]. Both observed and relative survival were determined to detect potential differences between survival trends and to compare the cantonal estimates with Swiss national [24] and international survival estimates.

Royston-Parmar survival models were fitted, using the patient’s survival time as outcome variable. Covariates in the models were incidence year (2018/2019, 2020, 2021), incidence month grouped into two-month intervals (Jan.-Feb., Mar.-Apr., May-Jun., Jul.-Aug., Sep.-Oct., Nov.-Dec.), patient’s sex (male, female; not included in the model for female breast and prostate cancer), age group (< 60, 60—69, 70—79, ≥ 80 years of age), cancer stage (I to IV or unknown), and canton of residence (ZH, ZG), all as categorical variables. An interaction term between incidence year and month (two-month intervals) was added. Baseline log-cumulative hazard was modelled as restricted cubic splines of the log-survival time with three degrees of freedom. By including the incidence year as continuous variable, evidence for a trend over the study period was explored. Modelling observed survival of cancer patients captures the direct and indirect impacts of the pandemic and provides directly interpretable estimates for key stakeholders, such as healthcare institutions, policymakers, and patients.

A counterfactual analysis was conducted, assuming that the pandemic had not happened. For cases diagnosed in 2020 and 2021, one- and three-year survival were estimated using a survival model that was based on data of patients diagnosed in 2018/2019 and included the incidence year as continuous variable. By using bootstrap resampling (B = 1000) with a sample size equal to the number of patients diagnosed in 2020 and 2021, respectively, the difference between average counterfactual and observed survival probability was calculated. Subsequently, mean survival difference and the 2.5th and 97.5th percentile of the bootstrap distribution, representing the 95% confidence interval (95% CI), were determined. Negative differences indicated a higher observed survival probability than predicted, while positive differences indicated the contrary.

All analyses were conducted using the software R (version 4.5.2 [39]) using the packages relsurv [40], survival [41], and rstpm2 [42] for the survival analyses. Statistical significance level was defined as a two-sided *P*-value of 0.05.

## Results

Overall, 32 826 cancer cases were included, of which 9.4% were cases of lung cancer, 9.4% colorectal cancer, 8.9% skin melanoma, 14.5% female breast cancer, 15.9% prostate cancer, and 41.9% belonged to tumor groups not analyzed separately (Table 1). The proportion of affected males was larger than of females for all-cancer (52.6% vs 47.4%), colorectal cancer (51.2% vs 48.8%), lung cancer (56.2% vs 43.8%), and skin melanoma (53.8% vs 46.2%). Median age at diagnosis was highest for colorectal and lung cancer (71.0 years), and lowest for female breast cancer (63.0). Among all cancer groups, between 90.5% and 93.1% of cases were residing in the more populous canton of ZH. Cases were similarly distributed across the incidence periods, except for prostate cancer with proportionally more cases in 2021 (28.5%) than in 2020 (23.9%) and 2018/2019 (47.6%).

**Table 1:** Characteristics of the study population including cancer cases diagnosed between 2018 and 2021 among residents of the Swiss Cantons of Zurich and Zug for all-cancer (*n* = 32 826), colorectal cancer (*n* = 3096), lung cancer (*n* = 3093), skin melanoma (*n* = 2918), female breast cancer (*n* = 4765), and prostate cancer (*n* = 5203)

Variables	All-cancer^a^	Colorectal Cancer^a^	Lung Cancer^a^	Skin Melanoma^a^	Female Breast Cancer^a^	Prostate Cancer^a^
n	32 826	3096	3093	2918	4765	5203
Sex, n (%)						
Males	17 267 (52.6%)	1586 (51.2%)	1737 (56.2%)	1569 (53.8%)	0 (0.0%)	5203 (100.0%)
Females	15,559 (47.4%)	1510 (48.8%)	1356 (43.8%)	1349 (46.2%)	4765 (100.0%)	0 (0.0%)
Age at Incidence (median [IQR])	68.0 [57.0, 77.0]	71.0 [59.0, 80.0]	71.0 [63.0, 77.0]	66.0 [52.0, 76.0]	63.0 [51.0, 74.0]	70.0 [64.0, 76.0]
Age Group at Incidence, n (%)						
< 60 years	9618 (29.3%)	779 (25.2%)	499 (16.1%)	1119 (38.3%)	2072 (43.5%)	721 (13.9%)
60–69 years	7743 (23.6%)	650 (21.0%)	929 (30.0%)	573 (19.6%)	990 (20.8%)	1715 (33.0%)
70–79 years	9388 (28.6%)	873 (28.2%)	1099 (35.5%)	719 (24.6%)	1022 (21.4%)	2032 (39.1%)
≥ 80 years	6077 (18.5%)	794 (25.6%)	566 (18.3%)	507 (17.4%)	681 (14.3%)	735 (14.1%)
Canton, n (%)						
Zurich	30 367 (92.5%)	2838 (91.7%)	2880 (93.1%)	2641 (90.5%)	4378 (91.9%)	4822 (92.7%)
Zug	2459 (7.5%)	258 (8.3%)	213 (6.9%)	277 (9.5%)	387 (8.1%)	381 (7.3%)
Incidence Year, n (%)						
2018/2019	16 385 (49.9%)	1568 (50.6%)	1565 (50.6%)	1453 (49.8%)	2404 (50.5%)	2479 (47.6%)
2020	7885 (24.0%)	731 (23.6%)	774 (25.0%)	690 (23.6%)	1132 (23.8%)	1243 (23.9%)
2021	8556 (26.1%)	797 (25.7%)	754 (24.4%)	775 (26.6%)	1229 (25.8%)	1481 (28.5%)

*IQR* Interquartile range

^a^Cancer cases were defined using the 10th revision of the international classification of diseases (ICD-10)[31]: All-cancer: all malignant cancers (except non-melanoma skin cancer ICD-10 C44) and benign brain cancer (ICD-10 D32—33, D43); Colorectal cancer: ICD-10 C18-C20; Lung cancer: ICD-10 C34; Skin melanoma: ICD-10 C43; Female breast cancer: ICD-10 C50; Prostate cancer: ICD-10 C61

Across all incidence years, observed and relative one- and three-year survival were highest for skin melanoma, and lowest for lung cancer (Table 2). Observed and relative one-year survival for all-cancer increased continuously from 82.2% [95% CI: 81.4%, 83.0%] and 83.8% [83.0%, 84.6%] in 2018 to 84.0% [83.3%, 84.8%] and 85.6% [84.9%, 86.4%] in 2021. Likewise, a steady increase in observed one-year survival was detected for prostate cancer (94.7% [93.5%, 95.9%] in 2018 to 96.9% [96.1%, 97.7%] in 2021). Observed and relative three-year survival for all-cancer continuously increased as well from 70.3% [69.4%, 71.3%] and 74.5% [73.5%, 75.5%] in 2018 to 72.9% [72.0%, 73.8%] and 77.4% [76.4%, 78.4%] in 2021. For three-year observed and relative survival, a U-shaped trend was observed from 2019 to 2021 for lung cancer, skin melanoma, female breast cancer, and prostate cancer, indicating a lower three-year survival for patients diagnosed during the first pandemic year.

**Table 2:** Age-standardized observed and relative one-year and three-year survival for cancer cases diagnosed between 2018 and 2021 of patients residing in the Swiss Cantons of Zurich and Zug for all-cancer (*n* = 32 826), colorectal cancer (*n* = 3096), lung cancer (*n* = 3093), skin melanoma (*n* = 2918), female breast cancer (*n* = 4765), and prostate cancer (*n* = 5203)

	Observed one-year survival				Relative one-year survival			
	2018 % (95% CI)	2019 % (95% CI)	2020 % (95% CI)	2021 % (95% CI)	2018 % (95% CI)	2019 % (95% CI)	2020 % (95% CI)	2021 % (95% CI)
All-cancer^a^	82.2% (81.4–83.0)	82.4% (81.6–83.2)	82.7% (81.9–83.5)	84.0% (83.3–84.8)	83.8% (83.0–84.6)	84.0% (83.2–84.8)	84.3% (83.4–85.1)	85.6% (84.9–86.4)
Colorectal Cancer^a^	87.1% (85.0–89.2)	87.4% (85.2–89.6)	85.6% (83.2–88.0)	86.3% (84.1–88.7)	88.6% (86.5–90.8)	89.0% (86.8–91.3)	87.3% (84.9–89.8)	88.1% (85.8–90.5)
Lung Cancer^a^	60.3% (56.7–64.1)	56.6% (53.1–60.3)	57.8% (53.0–63.0)	62.7% (59.5–66.2)	61.2% (57.7–65.0)	57.4% (54.0–61.1)	58.7% (54.3–63.4)	63.7% (60.4–67.2)
Skin Melanoma^a^	96.3% (94.8–97.7)	96.6% (95.3–97.9)	96.9% (95.5–98.2)	97.2% (96.1–98.4)	98.5% (96.9–100.0)	98.4% (97.1–99.8)	99.2% (97.8–100.6)	99.2% (98.0–100.4)
Female Breast Cancer^a^	96.0% (94.8–97.2)	95.4% (94.1–96.7)	94.9% (93.5–96.3)	96.2% (95.1–97.3)	97.7% (96.5–99.0)	97.0% (95.7–98.3)	96.5% (95.1–98.0)	97.9% (96.8–99.1)
Prostate Cancer^a^	94.7% (93.5–95.9)	95.6% (94.5–96.7)	95.9% (94.8–97.0)	96.9% (96.1–97.7)	96.7% (95.4–97.9)	97.6% (96.4–98.7)	97.9% (96.8–99.1)	98.7% (97.9–99.6)

	Observed three-year survival				Relative three-year survival			
	2018 % (95% CI)	2019 % (95% CI)	2020 % (95% CI)	2021 % (95% CI)	2018 % (95% CI)	2019 % (95% CI)	2020 % (95% CI)	2021 % (95% CI)
All-cancer^a^	70.3% (69.4–71.3)	70.9% (70.0–71.9)	71.1% (70.1–72.0)	72.9% (72.0–73.8)	74.5% (73.5–75.5)	75.1% (74.1–76.2)	75.2% (74.2–76.3)	77.4% (76.4–78.4)
Colorectal Cancer^a^	72.1% (69.2–75.2)	73.7% (70.7–76.7)	74.4% (71.4–77.6)	74.3% (71.4–77.4)	76.7% (73.5–80.0)	78.0% (74.9–81.3)	79.1% (75.8–82.5)	79.2% (76.1–82.5)
Lung Cancer^a^	40.7% (37.2–44.5)	39.2% (36.0–42.8)	37.8% (33.2–43.2)	42.3% (39.0–45.8)	42.5% (38.9–46.4)	40.9% (37.5–44.6)	39.6% (35.2–44.5)	44.3% (40.9–48.0)
Skin Melanoma^a^	90.1% (87.8–92.4)	91.7% (89.8–93.6)	90.4% (88.2–92.6)	92.2% (90.4–94.0)	96.8% (94.2–99.5)	98.2% (96.1–100.4)	97.5% (95.0–100.1)	98.4% (96.4–100.5)
Female Breast Cancer^a^	88.4% (86.6–90.3)	88.6% (86.8–90.5)	87.1% (85.0–89.1)	87.8% (86.0–89.8)	93.2% (91.2–95.4)	93.7% (91.6–95.8)	92.1% (89.9–94.4)	93.3% (91.2–95.4)
Prostate Cancer^a^	87.9% (86.2–89.6)	89.7% (88.1–91.3)	87.0% (83.0–91.2)	90.0% (88.6–91.5)	93.7% (91.8–95.7)	95.9% (94.1–97.7)	93.1% (89.6–96.7)	95.7% (94.0–97.3)

*CI* Confidence Interval

^a^Cancer cases were defined using the 10th revision of the international classification of diseases (ICD-10)[31]: All-cancer: all malignant cancers (except non-melanoma skin cancer ICD-10 C44) and benign brain cancer (ICD-10 D32—33, D43); Colorectal cancer: ICD-10 C18-C20; Lung cancer: ICD-10 C34; Skin melanoma: ICD-10 C43; Female breast cancer: ICD-10 C50; Prostate cancer: ICD-10 C61

All-cancer cases in 2020 (HR: 0.93 [95% CI: 0.89, 0.96]) and 2021 (0.91 [0.87, 0.94]) and skin melanoma cases in 2021 (0.79 [0.62, 0.98]) had a lower hazard of death compared to those in 2018/2019 (Table 3). For colorectal cancer cases, we observed an increased hazard of death for those diagnosed in 2020 (1.17 [1.03, 1.32]) and 2021 (1.24 [1.09, 1.40]) compared to those diagnosed pre-pandemic. Despite these differences, the *P*-value for trend indicated no evidence for a linear trend over the study period for any of the investigated cancer group. Across all years, elevated hazards of death were observed in Mar.-Apr. for all-cancer (1.06 [1.02, 1.11]), female breast cancer (1.29 [1.09, 1.50]), and prostate cancer (1.39 [1.20, 1.60]) compared to Jan.-Feb. The interaction term indicated that the observed seasonal impact in Mar.-Apr. was lower for prostate cancer in 2021 compared to pre-pandemic (0.62 [0.42, 0.88]). In contrast to Jan.-Feb., lower hazards of death among lung cancer cases were observed in all other periods (Mar.-Apr.: 0.83 [0.75, 0.91]; May-Jun.: 0.85 [0.77, 0.94]; Jul.-Aug.: 0.88 [0.79, 0.97]; Sep.-Oct.: 0.84 [0.75, 0.93]; Nov.-Dec.: 0.82 [0.74, 0.91]). Only in Nov.-Dec., the seasonal impact was stronger in 2020 compared with 2018/2019 (1.44 [1.16, 1.77]). Lower hazards of death were also observed for female breast cancer cases in May-Jun. (0.83 [0.69, 0.98]) and prostate cancer cases in Nov.-Dec. (0.81 [0.68, 0.96]) compared to the Jan.-Feb. period. None of the corresponding interaction terms showed evidence for a difference.

**Table 3:** Royston-Parmar survival model^a^ estimates and 95% confidence intervals for all-cancer (*n* = 32 826), colorectal cancer (*n* = 3096), lung cancer (*n* = 3093), skin melanoma (*n* = 2918), female breast cancer (*n* = 4765), and prostate cancer (*n* = 5203) cases diagnosed between 2018/2019 and 2021 and residing in the Swiss Cantons of Zurich and Zug

	All-cancer^b^	Colorectal cancer^b^	Lung cancer^b^	Skin melanoma^b^	Female breast cancer^b^	Prostate cancer^b^
	HR [95% CI]					
Incidence Year						
2018/2019 (ref.)	1.00 -	1.00 -	1.00 -	1.00 -	1.00 -	1.00 -
2020	0.93 [0.89;0.96]	1.17 [1.03; 1.32]	0.97 [0.89; 1.05]	0.86 [0.69; 1.04]	0.95 [0.82; 1.10]	0.94 [0.82; 1.08]
2021	0.91 [0.87; 0.94]	1.24 [1.09; 1.40]	0.99 [0.91; 1.08]	0.79 [0.62; 0.98]	0.94 [0.80; 1.10]	0.93 [0.79; 1.07]

	All-cancer^b^	Colorectal cancer^b^	Lung cancer^b^	Skin melanoma^b^	Female breast cancer^b^	Prostate cancer^b^
	*P*-value for trend					
Incidence Year	0.07	0.18	0.89	0.44	0.76	0.66

	All-cancer^b^	Colorectal cancer^b^	Lung cancer^b^	Skin melanoma^b^	Female breast cancer^b^	Prostate cancer^b^
	HR [95% CI]					
Incidence Month						
Jan.-Feb. (ref.)	1.00 -	1.00 -	1.00 -	1.00 -	1.00 -	1.00 -
Mar.-Apr	1.06 [1.02; 1.11]	1.07 [0.93; 1.21]	0.83 [0.75; 0.91]	0.91 [0.69; 1.17]	1.29 [1.09; 1.50]	1.39 [1.20; 1.60]
May-Jun	0.97 [0.93; 1.02]	0.95 [0.82; 1.10]	0.85 [0.77; 0.94]	0.94 [0.74; 1.17]	0.83 [0.69; 0.98]	0.98 [0.84; 1.13]
Jul.-Aug	1.00 [0.96; 1.05]	0.93 [0.81; 1.06]	0.88 [0.79; 0.97]	0.98 [0.78; 1.23]	0.98 [0.82; 1.16]	0.97 [0.82; 1.14]
Sep.-Oct	0.99 [0.95; 1.03]	0.91 [0.79; 1.05]	0.84 [0.75; 0.93]	0.89 [0.70; 1.12]	0.97 [0.82; 1.14]	0.98 [0.82; 1.17]
Nov.-Dec	0.96 [0.91; 1.00]^c^	1.15 [0.99; 1.32]	0.82 [0.74; 0.91]	0.90 [0.68; 1.16]	1.06 [0.89; 1.26]	0.81 [0.68; 0.96]

	All-cancer^b^	Colorectal cancer^b^	Lung cancer^b^	Skin melanoma^b^	Female breast cancer^b^	Prostate cancer^b^
	HR [95% CI]					
Interaction Term Incidence Year x Month						
2020 × Mar.-Apr	1.05 [0.96; 1.15]	0.78 [0.56; 1.05]	1.05 [0.85; 1.27]	0.69 [0.28; 1.40]	1.05 [0.72; 1.47]	0.75 [0.51; 1.05]
2021 × Mar.-Apr	0.97 [0.88; 1.06]	0.72 [0.54; 0.93]	0.96 [0.78; 1.18]	1.23 [0.72; 1.94]	0.77 [0.52; 1.08]	0.62 [0.42; 0.88]
2020 × May-Jun	0.99 [0.90; 1.08]	0.75 [0.54; 1.00]^d^	1.08 [0.89; 1.31]	1.55 [0.96; 2.36]	1.38 [0.97; 1.87]	0.98 [0.70; 1.31]
2021 × May-Jun	1.05 [0.96; 1.15]	0.71 [0.51; 0.96]	1.02 [0.82; 1.24]	1.78 [0.95; 2.97]	1.03 [0.66; 1.52]	1.18 [0.84; 1.60]
2020 × Jul.-Aug	1.01 [0.92; 1.10]	0.94 [0.69; 1.24]	1.03 [0.83; 1.26]	1.24 [0.71; 1.98]	1.10 [0.75; 1.54]	1.21 [0.86; 1.63]
2021 × Jul.-Aug	1.02 [0.93; 1.12]	0.95 [0.70; 1.25]	0.95 [0.76; 1.17]	1.28 [0.76; 1.98]	1.21 [0.79; 1.76]	1.03 [0.70; 1.46]
2020 × Sep.-Oct	1.05 [0.96; 1.15]	1.08 [0.78; 1.45]	1.01 [0.81; 1.23]	1.70 [1.04; 2.58]	1.10 [0.77; 1.51]	1.14 [0.76; 1.62]
2021 × Sep.-Oct	1.07 [0.97; 1.17]	0.88 [0.62; 1.21]	1.13 [0.90; 1.39]	1.36 [0.70; 2.32]	1.17 [0.77; 1.69]	1.07 [0.70; 1.56]
2020 × Nov.-Dec	1.13 [1.03; 1.24]	0.93 [0.67; 1.26]	1.44 [1.16; 1.77]	0.83 [0.46; 1.37]	1.05 [0.70; 1.50]	0.96 [0.66; 1.35]
2021 × Nov.-Dec	0.95 [0.85; 1.05]	0.58 [0.40; 0.81]	0.87 [0.68; 1.08]	0.78 [0.33; 1.50]	0.97 [0.62; 1.42]	0.85 [0.54; 1.25]

*HR* Hazard Ratio, *CI* Confidence Interval

^a^The models were additionally adjusted for four categorical variables indicating the patient’s sex (male, female; not included in the model for female breast cancer and for prostate cancer), age group (< 60, 60—69, 70—79, ≥ 80 years of age), cancer stage (I to IV or unknown), and canton of residence (ZH, ZG). An interaction term between incidence year and incidence month (two-month intervals) was included in all models. The baseline log-cumulative hazard was modelled as restricted cubic splines of the log-survival time with three degrees of freedom

^b^Cancer cases were defined using the 10th revision of the international classification of diseases (ICD-10)[31]: All-cancer: all malignant cancers (except non-melanoma skin cancer ICD-10 C44) and benign brain cancer (ICD-10 D32—33, D43); Colorectal cancer: ICD-10 C18-C20; Lung cancer: ICD-10 C34; Skin melanoma: ICD-10 C43; Female breast cancer: ICD-10 C50; Prostate cancer: ICD-10 C61

^c^0.9556 [0.9124; 1.0002]

^d^0.747 [0.543; 0.996]

**Fig. 1:**
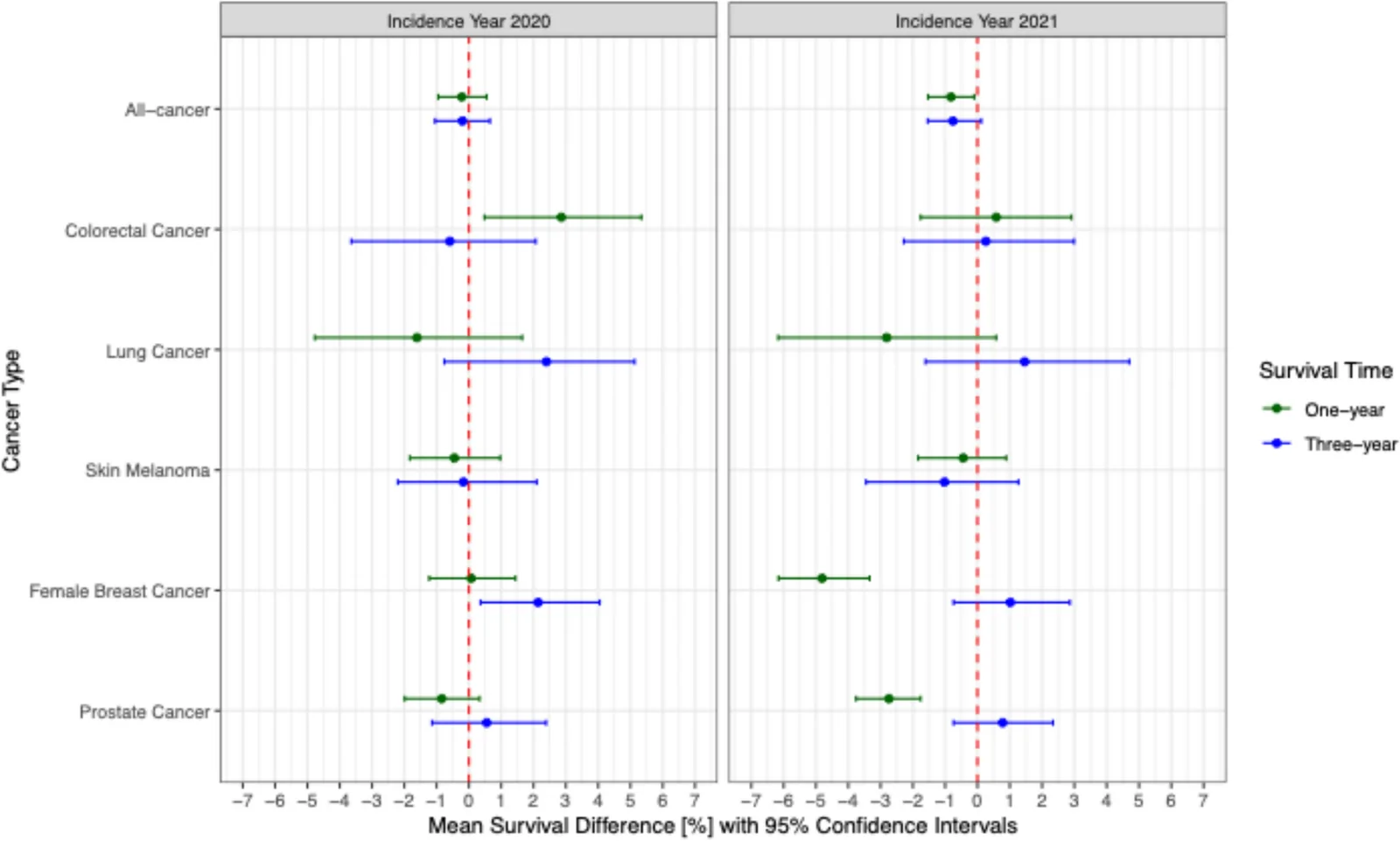
Mean difference between counterfactual and observed one-year and three-year survival probability for all-cancer (2020: *n* = 7885; 2021: *n* = *8556*), colorectal cancer (2020: *n* = 731; 2021: *n* = 797), lung cancer (2020: *n* = 774; 2021: *n* = 754), skin melanoma (2020: *n* = 690; 2021: *n* = 775), female breast cancer (2020: *n* = 1132; 2021: *n* = 1229), and prostate cancer (2020: *n* = 1243; 2021: *n* = 1481) cases diagnosed between 2020 and 2021 and residing in the Swiss Cantons of Zurich and Zug. Negative differences indicate a higher observed than predicted survival probability based on the counterfactual model, while positive differences indicate a lower observed than predicted survival probability based on the counterfactual model. The red dashed line indicates the reference value for no difference

In the counterfactual analysis (Fig. 1, Table S1), a negative mean difference, indicating higher observed than predicted survival, was estimated for the one-year survival of all-cancer (−0.82% [−1.53%, −0.10%]), female breast cancer (−4.81% [−6.16%, −3.34%]), and prostate cancer (−2.74% [−3.76%, −1.76%]) cases in 2021. A positive mean difference, indicating lower observed than predicted survival, was estimated for the one-year survival of colorectal cancer cases in 2020 (2.86% [0.48%, 5.34%]) and the three-year survival of female breast cancer cases in 2020 (2.14% [0.36%, 4.05%]).

## Discussion

This study used population-based data of 32 826 cancer cases diagnosed between 2018 and 2021 and registered at the CRZZSS. No clear patterns of higher mortality or worse survival during the pandemic compared to pre-pandemic years were observed. One- and three-year survival for all-cancer continuously increased over the study period. Three-year survival for lung, skin melanoma, female breast, and prostate cancer tended to be lower in 2020 compared to 2019 or 2021. Compared to pre-pandemic, the hazard of death was lower for all-cancer during the pandemic and skin melanoma in 2021, while it was higher for colorectal cancer during the pandemic. In 2021, higher observed than predicted one-year survival were estimated for all-cancer, female breast cancer, and prostate cancer, while the contrary was estimated for one-year survival of colorectal cancer and three-year survival of female breast cancer in 2020.

One- and three-year all-cancer survival continuously increased over the study period, reflecting the robustness and resilience of the health care system and the minimal impact of the COVID-19 pandemic on short- and intermediate-term all-cancer survival. Aligning with our findings, comparable one-year survival in pre-pandemic years and in 2020 and a modest increase in 2021 were reported based on national cancer registry data [24]. In contrast to the findings in Switzerland, observed one-year all-cancer survival in Northern Ireland was lower in 2020 (69.8% [68.6%, 70.9%]) compared to 2018/2019 (73.7% [72.9%, 74.4%]) [43]. A study including data from Denmark, Finland, Iceland, Norway, and Sweden found reduced one-year relative survival among patients in all countries except Iceland who were diagnosed between March and December 2020 compared to those diagnosed during the same period in 2011—2019 [44]. Older patients and those with aggressive tumors showed the largest reductions in one-year relative survival. [44] Lower relative one-year all-cancer survival was observed in the US in the second quarter of 2020 (77.5% [77.2%, 77.7%]) compared to 2018 (82.3% [82.1%, 82.5%]), but survival recovered already in the third quarter of 2020 [45].

Observed one-year prostate cancer survival increased from 2018 to 2021, reflecting a long-term trend most probably due to earlier diagnosis and improvements in prostate cancer screening and treatment [46]. In contrast, no meaningful difference in short-term prostate cancer survival before compared to during the pandemic has been observed in Northern Ireland[43], the US[45], and the UK[47].

A slight drop in three-year survival in 2020 for lung cancer, skin melanoma, female breast cancer, and prostate cancer were noted, which might either reflect annual variation or a potential impact of the pandemic. No difference in three-year survival in 2020 compared to pre-pandemic for lung cancer, skin melanoma, female breast cancer, and prostate cancer was reported in Belgium[48, 49], breast cancer in Taiwan[50], and colorectal cancer in Turkey[51].

Hazards of death were lower for all-cancer in 2020 and 2021 and skin melanoma in 2021 compared to 2018/2019, while for colorectal cancer the contrary was observed. In Mar.-Apr. compared to Jan.-Feb., higher hazards of death for all-cancer, female breast cancer, and prostate cancer were found, while the hazard of death was lower for lung cancer. An increasing treatment efficacy, earlier diagnosis, and a general trend towards increasing cancer survival over time might have led to the observed beneficial associations [52]. Although no major shift in the relative distribution of major cancer types across incidence years was observed, apart from a slight increase of prostate cancer diagnoses in 2021, potential case-mix bias cannot be fully excluded and may have had a minor influence on all-cancer survival estimates. For lung cancer in specific, patients with poor lung cancer prognosis may have been more vulnerable to COVID-19 and may have died before receiving a lung cancer diagnosis, leading to lower hazards of death [53]. Another explanation may be the higher rates of respiratory diseases and influenza typically observed during the winter months [54], which may delay lung cancer diagnosis due of overlapping symptoms and reduce cancer treatment tolerance [24, 43]. Meanwhile, the higher hazard of death during the pandemic, especially during the lockdown period in spring 2020, could have been due to the more limited healthcare capacity and changes in general health-seeking behavior, leading to non-adherence or modifications to the treatment plan and delayed diagnoses [10, 23, 24, 55, 56]. In addition, holidays such as the Easter break in March and April may have led to a reduction in healthcare utilization by people with mild symptoms and early-stage cancer, potentially resulting in a relative increase in more advanced cancer diagnoses with generally poorer prognosis [57]. Seasonal effects which have been observed inconsistently among cancer types might reflect random variation caused by multiple comparisons and small sample sizes.

In the counterfactual analysis, observed one-year survival in 2021 was higher than predicted for all-cancer, female breast cancer, and prostate cancer, but lower for one-year survival for colorectal cancer and three-year survival for female breast cancer in 2020. These results may indicate an adverse impact of the pandemic on cancer type-specific survival of patients diagnosed during the first year of the pandemic, while the impact already vanished during the second pandemic year. Similarly, a counterfactual analysis using the Surveillance, Epidemiology, and End Results data from the US estimated slightly more deaths among all-cancer (0.9%), colorectal (1.8%), female breast (0.4%), and prostate cancer (0.5%) patients and fewer deaths among lung (1.3%) and pancreas cancer (1.8%) patients diagnosed in 2020 than expected based on data from patients diagnosed in 2018 [58]. In the counterfactual analysis conducted by Lambert et al., one-year survival of patients diagnosed during compared to pre-pandemic did not differ for ten different cancer types in Canada [53]. Only patients aged 50–74 years with a lung cancer diagnosis between April to June 2021 had lower one-year survival than the corresponding pre-pandemic cohort (delta restricted mean survival times: − 31.6 days, 95% CI: − 58.3 to − 7.2 days) [53].

Our study had several strengths. The findings were based on population-based data of high quality, including two pre-pandemic years and the first two years of the pandemic. Not only the potential impact of the pandemic on short- but also on intermediate-term survival of overall cancer and of the five most common cancer types were explored. Our findings on one-year survival aligned with those reported by a study using Swiss national cancer registry data, implying that our observations on three-year cancer survival might be applicable to the general Swiss population. Our analyses were adjusted for known confounders, minimizing potential residual confounding. Lastly, by age-standardizing the survival rates, comparisons across time periods and with studies using data from other countries were possible. Despite these strengths, our study had some limitations. The results were based on data of only two Swiss cantons, potentially limiting thereby the generalizability of our study results to the whole country and resulting in a sample size that limits stratified analyses by age group or cancer stage. Data on the cause of death were not available. Hence, only all-cause mortality and no cause-specific hazards of deaths were investigated. The potential impact of the COVID-19 pandemic and the Cancer Registration Act could not be disentangled due to their concurrent occurrence in 2020.

To conclude, the COVID-19 pandemic appears to have had only a minimal impact on the general trend of increasing cancer survival in Switzerland. Our study findings provide important insights for policy makers and healthcare systems for future pandemics. Further studies are needed, including additional Swiss regions, a longer follow-up period, stratified analyses by age group or cancer stage, and cause of death information to enrich our knowledge of the multifaceted influences of the COVID-19 pandemic on cancer patients’ outcomes.

## Supplementary Information

Below is the link to the electronic supplementary material.

Supplementary file1 (DOCX 230 KB)

## Data Availability

The datasets generated during and/or analyzed during the current study are not publicly available since they are stored with personal identifiers but are available from the corresponding author upon reasonable request.

## Abbreviations

COVID-19: Coronavirus Disease 2019
WHO: World Health Organization
ZH: Zurich
ZG: Zug
SH: Schaffhausen
SZ: Schwyz
CI: Confidence Intervals
RECORD: REporting of studies Conducted using Observational Routinely-collected Data
CRZZSS: Cancer Registry of the Cantons Zurich, Zug, Schaffhausen, and Schwyz
ICD-10: International Classification of Diseases (10th revision)
IQR: Interquartile Range
DCO: Death Certificate Only

## Declarations

### Use of artificial intelligence (AI) tools

No AI tools were used.
