# Supplementary material for: Impact of the COVID-19 pandemic and its measures on one-year and three-year cancer survival using Swiss cantonal cancer registry data
Source: Cancer Causes Control. 2026 May 26;37(6):91. doi: 10.1007/s10552-026-02175-8 (PMC13668927; doi:10.1007/s10552-026-02175-8)
Supplement: Supplementary file 1 [file 10552_2026_2175_MOESM1_ESM.docx]

**Supplementary Material:**

**Impact of the COVID-19 pandemic and its measures on one-year and three-year cancer survival using Swiss cantonal cancer registry data**

**Authors**

Flurina Suter^1,2^, Miriam Wanner^2^, Andreas Wicki^3^, Dimitri Korol^2^, Sabine Rohrmann^1,2,*^

**Affiliation**

^1^ Division of Chronic Disease Epidemiology, Epidemiology, Biostatistics and Prevention Institute (EBPI), University of Zurich, Zurich, Switzerland

^2^ Cancer Registry Zurich, Zug, Schaffhausen and Schwyz, Division of Medical Oncology and Hematology, University Hospital Zurich, Zurich, Switzerland

^3^ Department of Medical Oncology and Hematology, Faculty of Medicine, University and University Hospital Zurich, Zurich, Switzerland

*Correspondence: Sabine Rohrmann, sabine.rohrmann@uzh.ch; Tel.: +41-446-345-256

**ORCID**

Flurina Suter: 0000-0002-5302-2268

Miriam Wanner: 0000-0003-0888-0690

Andreas Wicki: 0000-0002-2924-8080

Dimitri Korol: 0000-0002-9217-8038

Sabine Rohrmann: 0000-0002-2215-1200

**Journal name**

Cancer Causes & Control

# **Figure S1:** Flowchart of the study population from 2018-2021 of the Swiss cantons of Zurich and Zug^a^.

**Source Population:**

Malignant tumors (except C44) and benign brain tumors (D32-33, D43) diagnosed between 2018 and 2021 among residents of the Cantons of Zurich and Zug:

*n* = 34,933

**Exclusion Criteria:**

- Not first primary cancer diagnosis of a patient within the study period, 2018-2021 (*n* = 1316)
- Multiple first primary cancer diagnoses of a patient on the same day (*n* = 217)
- Death Certificate Only (DCO) cases (*n* = 227)
- Cases with an inaccurate incidence date (*n* = 347)

**Study Population^b^:**

All-cancer: *n* = 32,826

Colorectal Cancer: *n* = 3096

Lung Cancer: *n* = 3093

Skin Melanoma: *n* = 2918

Female Breast Cancer: *n* = 4765

Prostate Cancer: *n* = 5203

^a^ Cancer cases were classified using the 10^th^ revision of the international classification of diseases (ICD-10)[1]: All-cancer: all malignant cancers (except non-melanoma skin cancer ICD-10 C44) and benign brain cancer (ICD-10 D32-33, D43); Colorectal cancer: ICD-10 C18-C20; Lung cancer: ICD-10 C34; Skin melanoma: ICD-10 C43; Female breast cancer: ICD-10 C50; Prostate cancer: ICD-10 C61.

^b^ The study population consisted of cancer cases with one of the following ICD-10 codes[1]: C00-C26, C30-C34, C37-C41, C43, C45-C58, C60-C76, C80-C86, C88, C90-C96, D32-D33, D43.

**Table S1:** Estimates of the counterfactual analysis on one- and three-year survival among all-cancer (2020: *n* = 7885; 2021: *n = 8556*), colorectal cancer (2020: *n* = 731; 2021: *n =* 797), lung cancer (2020: *n* = 774; 2021: *n =* 754), skin melanoma (2020: *n* = 690; 2021: *n =* 775), female breast cancer (2020: *n* = 1132; 2021: *n =* 1229), and prostate cancer (2020: *n* = 1243; 2021: *n =* 1481) patients diagnosed during the first two years of the COVID-19 pandemic (2020-2021) and residing in the Swiss Cantons of Zurich and Zug.^a^

|  | **All-cancer^c^** | **Colorectal**  **Cancer^c^** | **Lung**  **Cancer^c^** | **Skin Melanoma^c^** | **Female Breast Cancer^c^** | **Prostate Cancer^c^** |
| --- | --- | --- | --- | --- | --- | --- |
| **N** |  |  |  |  |  |  |
| 2020 | 7885 | 731 | 774 | 690 | 1132 | 1243 |
| 2021 | 8556 | 797 | 754 | 775 | 1229 | 1481 |
|  |  |  |  |  |  |  |
| **One-year survival difference [%]**,  mean [95% CI]^b^ |  |  |  |  |  |  |
| 2020 | -0.22  [-0.95; 0.55] | 2.86  [0.48; 5.34] | -1.61  [-4.77; 1.65] | -0.45  [-1.83; 0.97] | 0.07  [-1.23; 1.44] | -0.84  [-1.99; 0.33] |
| 2021 | -0.82  [-1.53; -0.10] | 0.58  [-1.76; 2.91] | -2.81  [-6.17; 0.58] | -0.44  [-1.84; 0.89] | -4.81  [-6.16; -3.34] | -2.74  [-3.76; -1.76] |
|  |  |  |  |  |  |  |
| **Three-year survival difference [%]**,  mean [95% CI]^b^ |  |  |  |  |  |  |
| 2020 | -0.20  [-1.05; 0.65] | -0.59  [-3.64; 2.07] | 2.40  [-0.76; 5.13] | -0.17  [-2.20; 2.10] | 2.14  [0.36; 4.05] | 0.55  [-1.14; 2.39] |
| 2021 | -0.76  [-1.54; 0.12] | 0.26  [-2.28; 2.99] | 1.46  [-1.61; 4.70] | -1.02  [-3.45; 1.27] | 1.02  [-0.74; 2.86] | 0.78  [-0.74; 2.34] |

CI = Confidence Interval.

^a^ The counterfactual survival probability among patients diagnosed in 2020 and 2021 was based on a Royston Parmar survival model using only the data of patients diagnosed in 2018 and 2019 and adjusting for the continuous variable indicating the incidence year (2018, 2019) and the categorical variables indicating the incidence month (two-month intervals: Jan.-Feb., Mar.-Apr., May-Jun., Jul.-Aug., Sep.-Oct., Nov.-Dec.), the patient’s sex (male, female; not included in the model for female breast cancer and for prostate cancer), age group (<60, 60-69, 70-79, ≥80 years of age), cancer stage (I to IV or unknown), and canton of residence (ZH, ZG). Additionally, the model included an interaction term between incidence year and incidence month (two-month intervals).

^b^ By using bootstrap resampling (B = 1000) with a sample size equal to the number of patients diagnosed in 2020 and 2021, respectively, the difference between average counterfactual survival probability and observed survival probability was calculated. Subsequently, the average survival difference and the 2.5th and 97.5th percentile, representing the 95% confidence interval of the bootstrap distribution were determined. Negative differences indicated a higher observed than predicted survival probability based on the counterfactual model, while positive differences indicated a lower observed than predicted survival probability based on the counterfactual model.

**^c^** Cancer cases were defined using the 10^th^ revision of the international classification of diseases (ICD-10) [1]: All-cancer: all malignant cancers (except non-melanoma skin cancer ICD-10 C44) and benign brain cancer (ICD-10 D32-33, D43); Colorectal cancer: ICD-10 C18-C20; Lung cancer: ICD-10 C34; Skin melanoma: ICD-10 C43; Female breast cancer: ICD-10 C50; Prostate cancer: ICD-10 C61.

**References**

[1]	World Health Organization. ICD-10 version: 2019 - International statistical classification of diseases and related health problems 10th revision 2019. https://icd.who.int/browse10/2019/en (accessed November 18, 2022).
